# High density lipoprotein promotes proliferation of adipose-derived stem cells via S1P1 receptor and Akt, ERK1/2 signal pathways

**DOI:** 10.1186/s13287-015-0090-5

**Published:** 2015-05-15

**Authors:** Haitao Shen, Enchen Zhou, Xiujing Wei, Zhiwei Fu, Chenguang Niu, Yang Li, Bing Pan, Anna V Mathew, Xu Wang, Subramaniam Pennathur, Lemin Zheng, Yongyu Wang

**Affiliations:** Department of Pathology, Shantou University Medical College, Shantou, Guangdong 515041 China; Department of Neurosurgery, The First Affiliated Hospital of Soochow University, Suzhou, 215006 China; The Institute of Cardiovascular Sciences and Institute of Systems Biomedicine, School of Basic Medical Sciences, Key Laboratory of Molecular Cardiovascular Sciences of Education Ministry, Peking University Health Science Center, Beijing, 100191 China; Hutchison Medi Pharma (Suzhou), Jiangsu, 215125 China; Department of Medicine, University of Michigan, Ann Arbor, MI 48109 USA; Institute of Hypoxia Medicine, Wenzhou Medical University, Zhejiang, 325035 China

## Abstract

**Introduction:**

Adipose-derived stem cells (ADSC) are non-hematopoietic mesenchymal stem cells that have shown great promise in their ability to differentiate into multiple cell lineages. Their ubiquitous nature and the ease of harvesting have attracted the attention of many researchers, and they pose as an ideal candidate for applications in regenerative medicine. Several reports have demonstrated that transplanting ADSC can promote repair of injured tissue and angiogenesis in animal models. Survival of these cells after transplant remains a key limiting factor for the success of ADSC transplantation. Circulating factors like High Density Lipoprotein (HDL) has been known to promote survival of other stems cells like bone marrow derived stem cells and endothelial progenitor cells, both by proliferation and by inhibiting cell apoptosis. The effect of HDL on transplanted adipose-derived stem cells *in vivo* is largely unknown.

**Methods:**

This study focused on exploring the effects of plasma HDL on ADSC and delineating the mechanisms involved in their proliferation after entering the bloodstream. Using the MTT and BrdU assays, we tested the effects of HDL on ADSC proliferation. We probed the downstream intracellular Akt and ERK1/2 signaling pathways and expression of cyclin proteins in ADSC using western blot.

**Results:**

Our study found that HDL promotes proliferation of ADSC, by binding to sphingosine-1- phosphate receptor-1(S1P1) on the cell membrane. This interaction led to activation of intracellular Akt and ERK1/2 signaling pathways, resulting in increased expression of cyclin D1 and cyclin E, and simultaneous reduction in expression of cyclin-dependent kinase inhibitors p21 and p27, therefore promoting cell cycle progression and cell proliferation.

**Conclusions:**

These studies raise the possibility that HDL may be a physiologic regulator of stem cells and increasing HDL concentrations may be valuable strategy to promote ADSC transplantation.

**Electronic supplementary material:**

The online version of this article (doi:10.1186/s13287-015-0090-5) contains supplementary material, which is available to authorized users.

## Introduction

Adipose-derived stem cells (ADSCs) are multipotent, and hence can differentiate into a variety of cell types including adipocytes, chondrocytes, osteocytes, smooth muscle cells, and endothelial cells [1, 2]. This potential is the basis of their application in stem cell-based regenerative therapy for a variety of disorders [3]. Compared with bone marrow-derived stem cells, ADSCs offer an unparalleled advantage because they are widely available, easily harvested and proliferate rapidly *in vitro*. Autologous cells are also nonimmunogenic and their use does not raise ethical concerns, in contrast to embryonic stem cell use. Although they originate from the mesodermal lineage, the differentiating potential of ADSCs is not limited to mesodermal tissues [4]. In recent years, studies have confirmed that ADSCs transplanted into various animal models with organ or tissue injury can differentiate into vascular endothelial cells and play an essential role in injury repair through contributions to angiogenesis [5]. Transplantation of ADSCs is a novel therapeutic option for treatment in various cardiovascular diseases, particularly those involving vascular injury [3, 6]. Low survival rate of ADSCs after transplantation due to low proliferation and loss from apoptosis remains a significant problem in these cells [7]. The proliferating capacity of these cells is dependent on donor age, type of adipose tissue, harvesting procedure, culture conditions and several growth factors [8, 9].

The beneficial role of circulating plasma high density lipoprotein (HDL) in promoting proliferation of other stem cell lines, such as vascular endothelial precursor cells [10] and bone marrow-derived stem cells [11], is well established. HDL’s primary role is in mediating reverse cholesterol transport from the lipid-laden macrophages in the vascular wall back to the liver [12]. Clinical evidence supports the inverse association between cardiovascular disease and HDL cholesterol levels [13]. In addition to playing an important role in lipid metabolism, HDL possesses many antioxidative, anti-inflammatory and antithrombotic effects. The antioxidant effect of HDL is mainly reflected in the inhibition of low density lipoprotein oxidation, which is a critical inciting factor in the occurrence and evolution of atherosclerosis. HDL inhibits endothelial cell apoptosis, promotes endothelial cell repair and activation of endothelial nitric oxide synthase, and performs many other related anti-inflammatory functions [14].

Transplanted ADSCs are in contact with circulating HDL, but the effects of HDL on circulating ADSCs are still not clear. In this study, we propose that HDL promotes ADSC proliferation and regulates the physiology of ADSCs, thereby providing a therapeutic target to promote repair of vascular injury.

## Materials and methods

### Isolation, culture and identification of adipose-derived stem cells

All animal experimental procedures were approved by the Ethics Committee of Animal Research, Peking University Health Science Center, and the investigation conformed to the Guide for the Care and Use of Laboratory Animals published by the US National Institutes of Health (National Institutes of Health publication updated in 2011). Samples of adipose tissue were collected from the groins of 6-week-old ICR mice. The adipose tissue was then shredded into small pieces and digested with 10 mg/ml collagenase I (Sigma-Aldrich, St. Louis, MO, USA) for 1 hour at 37 °C. The digestion was terminated by adding Dulbecco’s modified Eagle’s medium (DMEM) medium (Hyclone, USA) containing 10 % fetal bovine serum (FBS; Hyclone, Logan, UT, USA) and 1 % penicillin/streptomycin (GIBCO, Gibco, part of Life Technologies, Carlsbad, CA, USA). The tissue suspension was centrifuged at 200 × *g* for 5 minutes and the pellet was suspended in fresh medium, and then plated into 100 mm dishes. Nonadherent cells were removed by replacing fresh medium after 24 hours. The cells were passaged following trypsin digestion when they reached 90 % confluence. After three passages, cells were harvested with 0.05 % trypsin digestion, washed three times with phosphate-buffered saline (PBS) and incubated with antibodies CD34-fluorescein isothiocyanate, CD45-fluorescein isothiocyanate, CD44-phycoerythrin (PE) and Sca1-PE (BD Biosciences, San Jose, CA, USA) at 37 °C for 30 minutes. The cells were then washed three times with PBS and suspended in 300 μl PBS, analyzed with flow cytometry and at least 10^4^ events per sample were recorded. CD29, CD90 and CD105 were also tested using APC anti-mouse CD29 (Miltenyi Biotec, Bergisch Gladbach, Germany), APC Arm hamster IgG Isotype Ctrl (eBioscience, San Diego, CA, USA), PE anti-rat CD90/mouse CD90.1 (Biolegend, San Diego, CA, USA), PE mouse IgG1,κ Isotype Ctrl (Biolegend, San Diego, CA, USA), PE anti-mouse CD105 (Biolegend, San Diego, CA, USA), and PE rat IgG2a,κ Isotype Ctrl (Biolegend, San Diego, CA, USA).

The human ADSCs were purchased from American Type Culture Collection (PCS-500-011; ATCC, USA). The cells were cultured in Mesenchymal Stem Cell Basal Medium (PCS-500-030; ATCC) with a Mesenchymal Stem Cell Growth Kit (PCS-500-040; ATCC) and 1 % penicillin/streptomycin (GIBCO), and were passaged after trypsinization when they reached 90 % confluence.

### Preparation of high density lipoprotein

HDL was prepared as described in our previous report [13]. Plasma samples were collected from healthy volunteers. The study protocol was approved by the Institutional Review Board of Peking University Health Science Center. Each participant gave written, informed consent after the nature of the procedure was explained. The plasma density was adjusted to 1.3 g/ml with KBr, and saline (1.006 g/ml) was layered over the adjusted plasma to form a discontinuous NaCl/KBr density gradient. The samples with gradient were centrifuged at 350,000 × *g* for 3.5 hours at 4 °C. The purity of HDL was evaluated by 12 % SDS-PAGE and western blot analysis using goat anti-apoA-I polyclonal antibody (DiaSorin, Stillwater, OK, USA) and was quantified through the measurement of apolipoprotein A-I content by nephelometry (Dimension XPand; Dade Behring, Marburg, Germany). HDL was dialyzed with PBS, sterilized and stored in the dark at 4 °C for use within 1 month.

### Treatment with HDL, cell signaling pathway inhibitor and sphingosine-1-phosphate type 1 receptor inhibitor

The ADSCs were passaged after trypsin digestion when they reached 90 % confluence in 100 mm dishes, and then plated into six-well plates. When they reached a confluence of 70 %, for mice ADSCs the medium was changed with fresh DMEM containing 1 % FBS, but for human ADSCs the medium was replaced with Mesenchymal Stem Cell Basal Medium (PCS-500-030; ATCC) containing 1 % FBS, and then exposed to HDL at different concentrations (0, 20, 50, 100, 200 μg/ml). To study the effects of the HDL temporally, the cells were exposed to 100 μg/ml HDL at different time points (0, 0.5, 1, 2, 4 hours). Before HDL treatment the cells were incubated with either Akt inhibitor (LY294002, 25 μM) or mitogen-activated protein kinase inhibitor (PD98059, 50 μM) for 2 hours or with sphingosine-1-phosphate type 1 (S1P1) receptor inhibitor (VPC23019, 15 μM) for 30 minutes in medium without FBS. After pretreatment, the cells were cultured with 100 μg/ml HDL for 1 hour in fresh DMEM containing 1 % FBS or Mesenchymal Stem Cell Basal Medium containing 1 % FBS respectively.

### MTT assay and bromodeoxyuridine assay

For the MTT assay, cells (3,000 cells/well) were plated into a 96-well plate and cultured overnight, and then the medium was replaced with fresh DMEM containing 1 % FBS or Mesenchymal Stem Cell Basal Medium containing 1 % FBS respectively. The cells were incubated in different concentrations of HDL (0, 20, 50, 100, 200 μg/ml) for 24 hours and treated with 100 μg/ml HDL at different time points (6, 12, 24, 48 hours), and then 10 μl MTT reagent was added to each well for an additional 4 hours. Dimethylsulfoxide (200 μl) was added to each well and then absorbance at 570 nm was measured using an enzyme-linked immunosorbent assay reader.

For bromodeoxyuridine (BrdU) assay (Roche, Mannheim, Germany), the cells are prepared similarly to those for the MTT assay. After incubation with HDL, 10 μl BrdU was added to the medium and incubated for an additional 4 hours. The cells were then fixed, and incubated with BrdU antibody for 60 minutes. After three washes, cells were incubated with 200 μl substrate solution for 5 to 30 minutes and 25 μl of 1 M H_2_SO_4_ was added. Finally, absorbance at 450 nm was measured using an enzyme-linked immunosorbent assay reader.

### Western blot analysis

ADSCs were washed three times with PBS, and lysed in RIPA lysis buffer on ice for 30 minutes. The lysates were centrifuged at 4 °C, 12,000 X g for 10 minutes. The supernatant was collected. Equal amounts of protein (40 μg) were separated by 12 % SDS-PAGE and transferred to nitrocellulose membranes. The membranes were blocked with 5 % nonfat milk in Tris-buffered saline/Tween 20 for 1 hour and incubated with primary antibody at 4 °C overnight, and then incubated with appropriate horseradish peroxidase-conjugated secondary antibody. The immune complexes were detected by electrochemiluminescence.

### Statistical analysis

Statistical analysis was performed with Graph Pad prism 5 software (version 5.01; GraphPad Software, La Jolla, CA, USA). All data are reported as mean ± standard error of the mean. The significant differences between means were evaluated using the unpaired *t* test. *P* <0.05 was considered a statistically significant difference.

## Results

### Characterization of mice adipose-derived stem cells

**Fig. 1:**
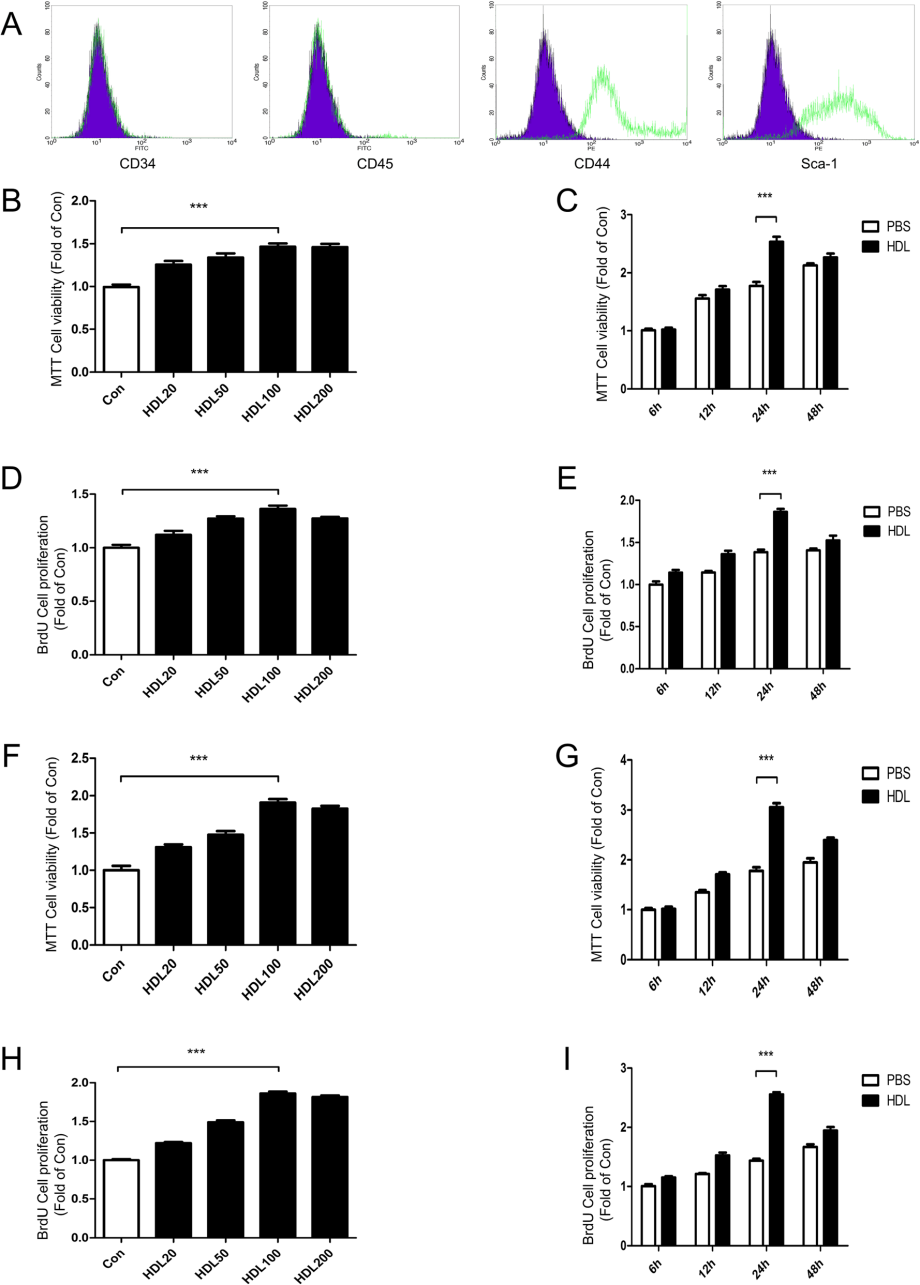
High density lipoprotein promotes proliferation of adipose-derived stem cells. **a** Characterization of mice adipose-derived stem cell (ADSC) markers: cells were labeled with fluorescein isothiocyanate (FITC)-conjugated and phycoerythrin (PE)-conjugated antibodies and evaluated by flow cytometry. After three passages, ADSCs were positive for CD44 and Sca-1, but negative for CD34 and CD45. Black lines, IgG isotype control. **b** MTT assay and **d** bromodeoxyuridine (BrdU) assay: cell proliferation in mice ADSCs treated with different concentrations (0, 20, 50, 100, 200 μg/ml) of high density lipoprotein (HDL) for 24 hours. **c** MTT assay and **e** BrdU assay: cell proliferation of mice ADSCs treated with HDL over various time periods (6 to 48 hours). **f** MTT assay and **h** BrdU assay: cell proliferation in human ADSCs treated with different concentrations (0, 20, 50, 100, 200 μg/ml) of HDL for 24 hours. **g** MTT assay and **i** BrdU assay: cell proliferation of human ADSCs over various time periods (6 to 48 hours). Bar graphs represent mean ± standard error of the mean, ***P <0.001. PBS, phosphate-buffered saline

The adherent cultured mice ADSCs display a fibroblast-like morphology and expand in a monolayer on tissue culture plates when observed under a light microscope. Flow cytometry was performed to confirm the phenotype of the adherent cells. The results showed that these cells are positive for CD44 (91.44 %) and Sca-1 (80.13 %) and negative for CD34 (0.68 %) and CD45 (1.50 %) (Fig. 1a). These results are consistent with previously published reports [4, 7] and confirm that the cells were mice ADSCs. CD29, CD90 and CD105 were also tested (Additional file 1), which also confirm that the cells were mice ADSCs.

### High density lipoprotein promotes adipose-derived stem cell proliferation

**Fig. 2:**
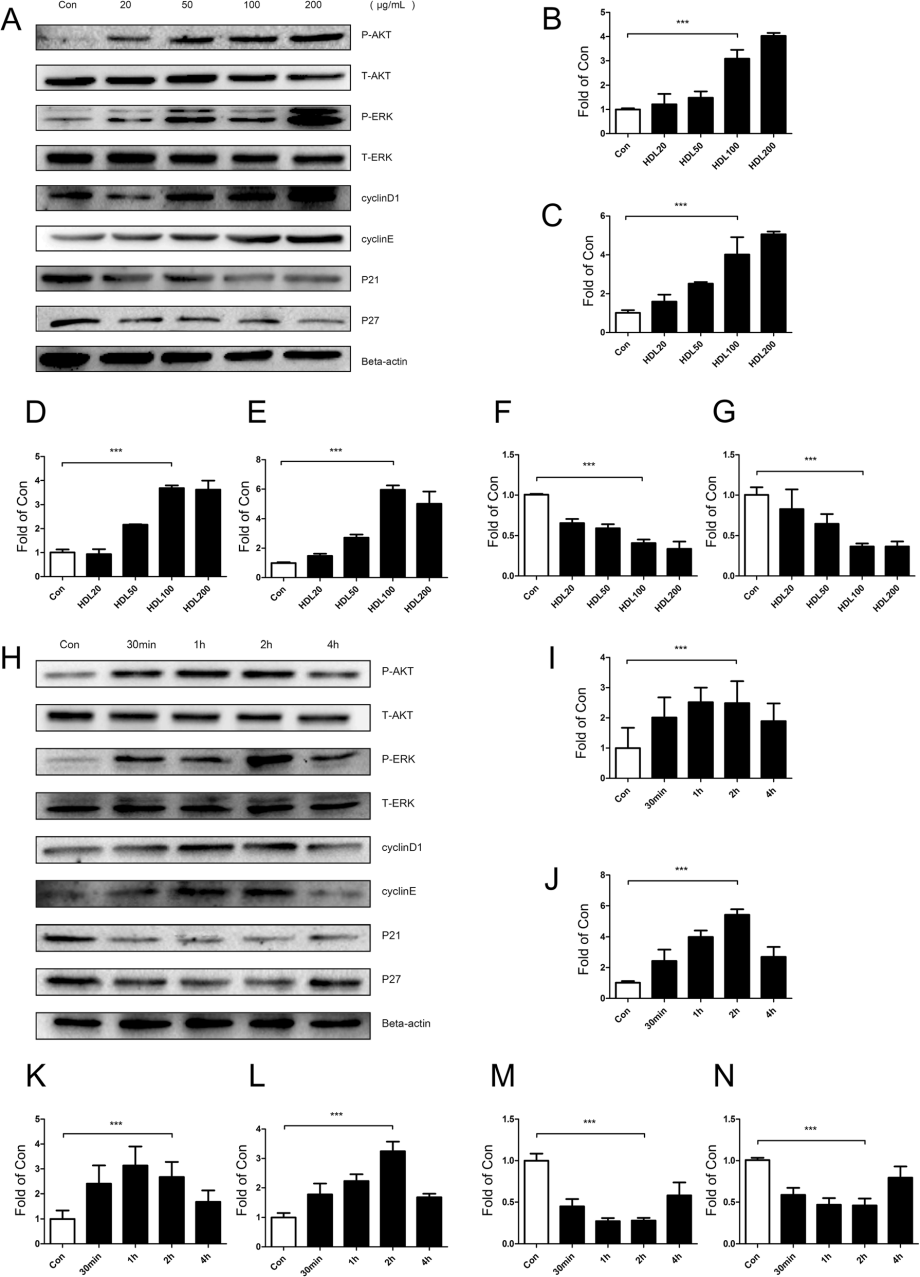
Akt and ERK1/2 signal pathways, cell cyclins (cyclin D_1_/E) and cyclin-dependent kinase inhibitors (p21/p27) are involved in high density lipoprotein promoting mice adipose-derived stem cell proliferation. Western blot expression of various signal pathway molecules and cell cycle regulatory proteins of mice adipose-derived stem cells which were treated with high density lipoprotein (HDL) at different concentrations **a** and at various time points **h**. **b** p-Akt, **c** p-ERK, **d** cyclin D_1_, **e** cyclin E, **f** p21, **g** p27, **i** p-Akt, **j** p-ERK, **k** cyclin D_1_, **l** cyclin E, **m** p21, **n** p27. Results are the mean ± standard error of the mean of three experiments for each condition determined by densitometry relative to corresponding total protein/beta-actin. *P <0.05, **P <0.01, ***P <0.001

**Fig. 3:**
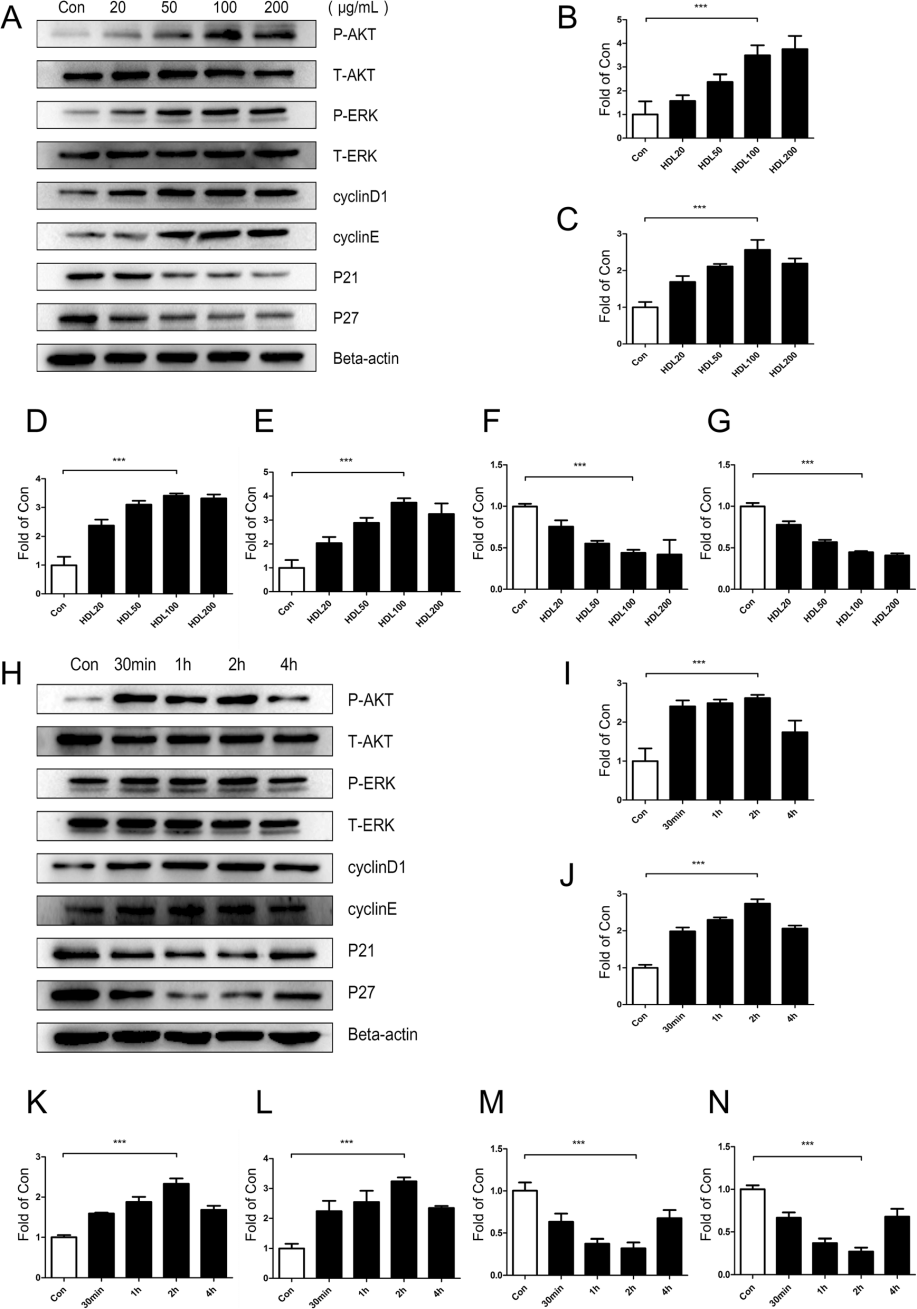
Akt and ERK1/2 signal pathways, cell cyclins (cyclin D_1_/E) and cyclin-dependent kinase inhibitors (p21/p27) are involved in high density lipoprotein promoting human adipose-derived stem cell proliferation. Western blot expression of various signal pathway molecules and cell cycle regulatory proteins of human adipose-derived stem cells which were treated with high density lipoprotein (HDL) at different concentrations **a** and at various time points **h**. **b** p-Akt, **c** p-ERK, **d** cyclin D_1_, **e** cyclin E, **f** p21, **g** p27, **i** p-Akt, **j** p-ERK, **k** cyclin D_1_, **l** cyclin E, **m** p21, **n** (p27). Results are the mean ± standard error of three experiments for each condition determined by densitometry relative to corresponding total protein/beta-actin. *P <0.05, **P <0.01, ***P <0.001

The proliferative effects of different HDL concentrations on ADSCs incubated with HDL for 24 hours were measured with MTT assay and BrdU incorporation assay. Our results indicate that there is a dose-dependent HDL-stimulated proliferation of ADSCs with the highest proliferation at a concentration of 100 or 200 μg/ml (Fig. 1b,d,f,h). Time-dependent effects of HDL in promoting proliferation of ADSCs were also tested. We found that the effect of HDL promoting ADSC proliferation reaches a maximum at 24 hours (Fig. 1c,e,g,i). Western blot results showed that phosphorylation of Akt and extracellular signal-related kinase (ERK)1/2 and expression of cyclin D_1_ and cyclin E were significantly increased, while the expression of cyclin-dependent kinase inhibitors (CKIs) p21 and p27 were reduced markedly (Figs. 2a,h and 3a,h).

### Akt and ERK1/2 signaling pathways play an essential role in HDL-induced proliferation of adipose-derived stem cells

**Fig. 4:**
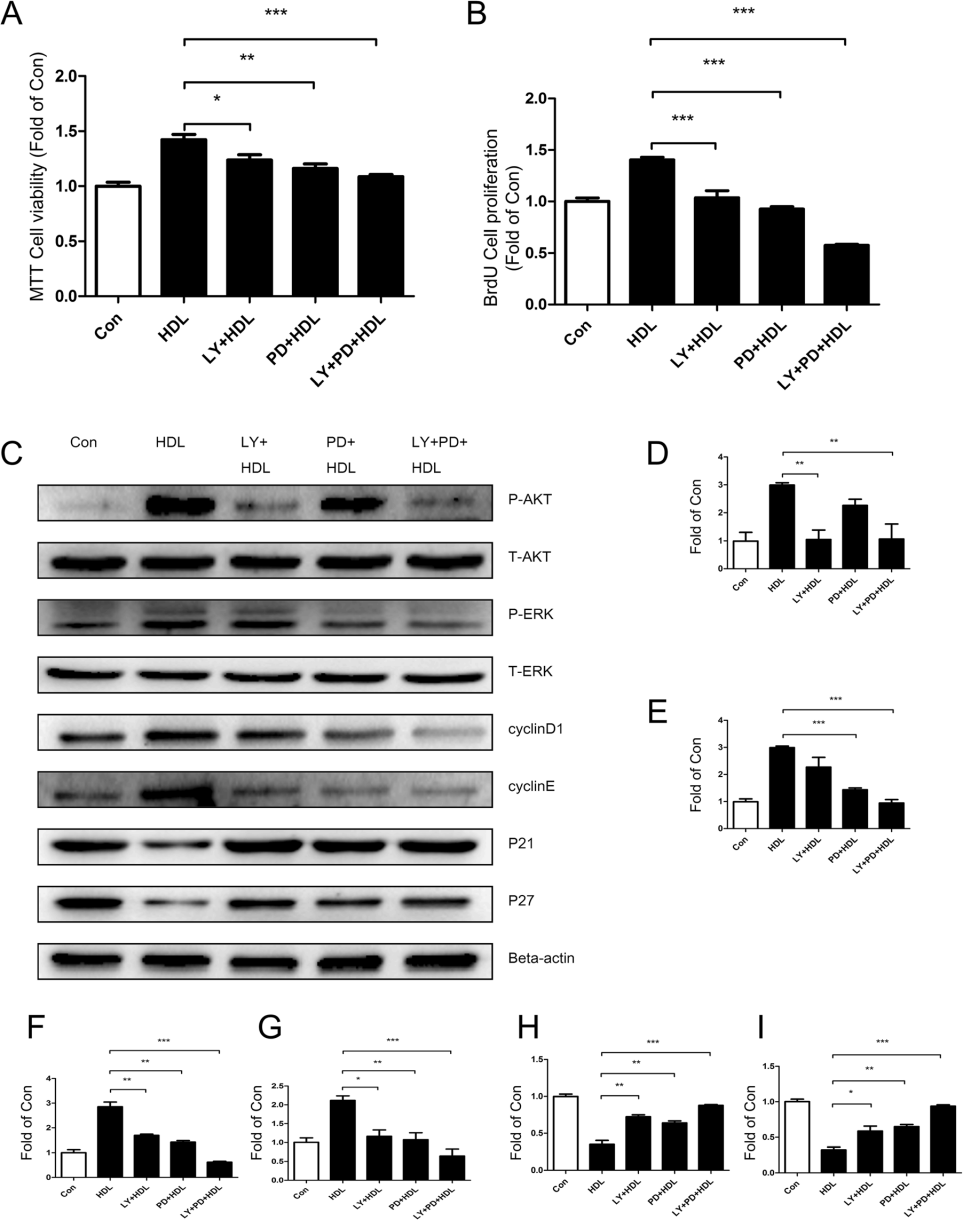
High density lipoprotein-mediated proliferation of mice adipose-derived stem cells is significantly decreased when treated with signal pathway inhibitors (LY294002 or/and PD98059). **a** MTT assay and **b** bromodeoxyuridine (BrdU) assay: mice adipose-derived stem cells (ADSCs) were pretreated LY249002 or/and PD98059 for 2 hours prior to 24-hour incubation with high density lipoprotein (HDL). Data show that the proliferation of mice ADSCs was obviously inhibited with LY249002 and PD98059 treatment. **c** Western blot change in expression of various signal pathway molecules and cell cycle regulatory proteins in mice ADSCs that were pretreated with LY249002 or/and PD98059 for 2 hours prior to 1-hour incubation with HDL. **d** p-Akt, **e** p-ERK, **f** cyclin D_1_, **g** cyclin E, **h** p21, **i** p27. Results are the mean ± standard error of three experiments for each condition determined by densitometry relative to corresponding total protein/beta-actin. *P <0.05, **P <0.01, ***P <0.001

**Fig. 5:**
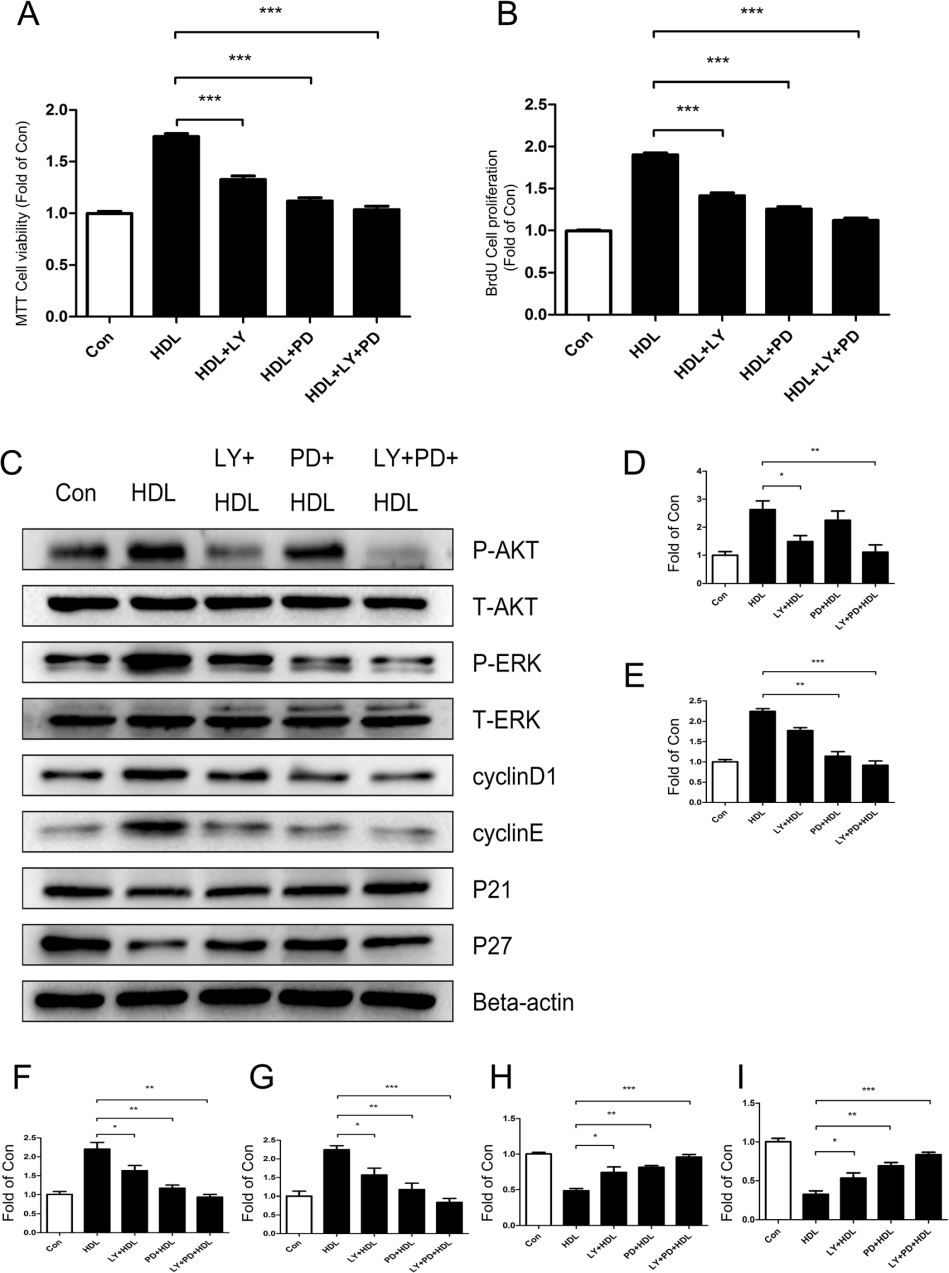
High density lipoprotein-mediated proliferation of human adipose-derived stem cells is significantly decreased when treated with signal pathway inhibitors (LY294002 or/and PD98059). **a** MTT and **b** bromodeoxyuridine (BrdU) assay: human mice adipose-derived stem cells (ADSCs) were pretreated with LY249002 or/and PD98059 for 2 hours prior to 24-hour incubation with high density lipoprotein (HDL). Data show that the proliferation of human ADSCs was obviously inhibited with LY249002 and PD98059 treatment. **c** Western blot change in expression of various signal pathway molecules and cell cycle regulatory proteins in human ADSCs that were pretreated with LY249002 or/and PD98059 for 2 hours prior to 1-hour incubation with HDL. **d** p-Akt, **e** p-ERK, **f** cyclin D_1_, **g** cyclin E, **h** p21, **i** p27. Results are the mean ± standard error of three experiments for each condition determined by densitometry relative to corresponding total protein/beta-actin. *P <0.05, **P <0.01, ***P <0.001

Pretreating ADSCs with Akt signaling pathway inhibitor LY294002 and ERK1/2 signaling pathway inhibitor PD98059 for 2 hours before incubation with HDL suppressed the HDL-stimulated ADSC proliferation (Figs. 4a,b and 5a,b). The results of western blot analysis also show that pretreatment with the two signaling pathway inhibitors blocked the effect of Akt and ERK1/2 activation, attenuated the HDL-induced increment of cell cyclins (cyclin D_1_/E) and increased the expression of CKIs (p21/p27). These results suggest that HDL required functional activation of Akt and ERK1/2 signal pathways to mediate its proliferative effect on ADSC (Figs. 4c and 5c).

### Involvement of S1P1 receptor in HDL-induced proliferation of adipose-derived stem cells

**Fig. 6:**
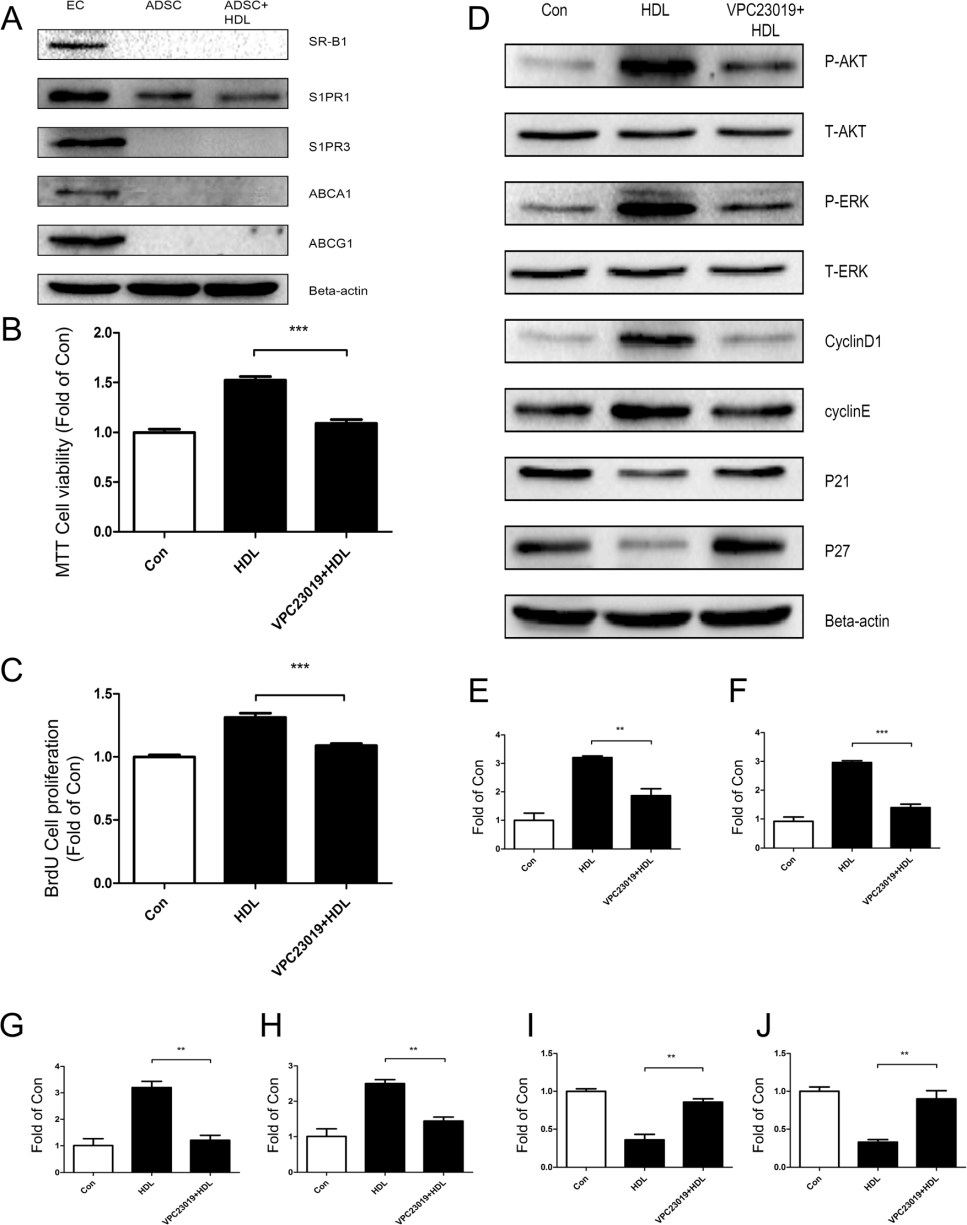
S1P1 receptor contributes to high density lipoprotein-induced cell proliferation of mice adipose-derived stem cells. **a** Western blot confirms the presence of several classical receptors for high density lipoprotein (HDL) in mice adipose-derived stem cells (ADSCs). Human umbilical vein endothelial cells (EC) were used as a positive control to validate the result. Data show that only S1P1, a classical receptor for HDL, was expressed in mice ADSCs. **b** MTT assay and **c** bromodeoxyuridine (BrdU) assay show that the effect of HDL-promoting cell proliferation in mice ADSCs was blocked when pretreated with S1P1 receptor specific inhibitor VPC23019 for 30 minutes prior to 24 hours of HDL treatment. **d** Western blot change in expression of various signal pathway molecules and cell cycle regulatory proteins in mice ADSCs pretreated with S1P1 receptor inhibitor VPC23019 for 30 minutes prior to incubation with HDL for 1 hour. **e** p-Akt, **f** p-ERK, **g** cyclin D_1_, **h** cyclin E, **i** p21, **j** p27. Results are the mean ± standard error of three experiments for each condition determined by densitometry relative to corresponding total protein/beta-actin. *P <0.05, **P <0.01, ***P <0.001

**Fig. 7:**
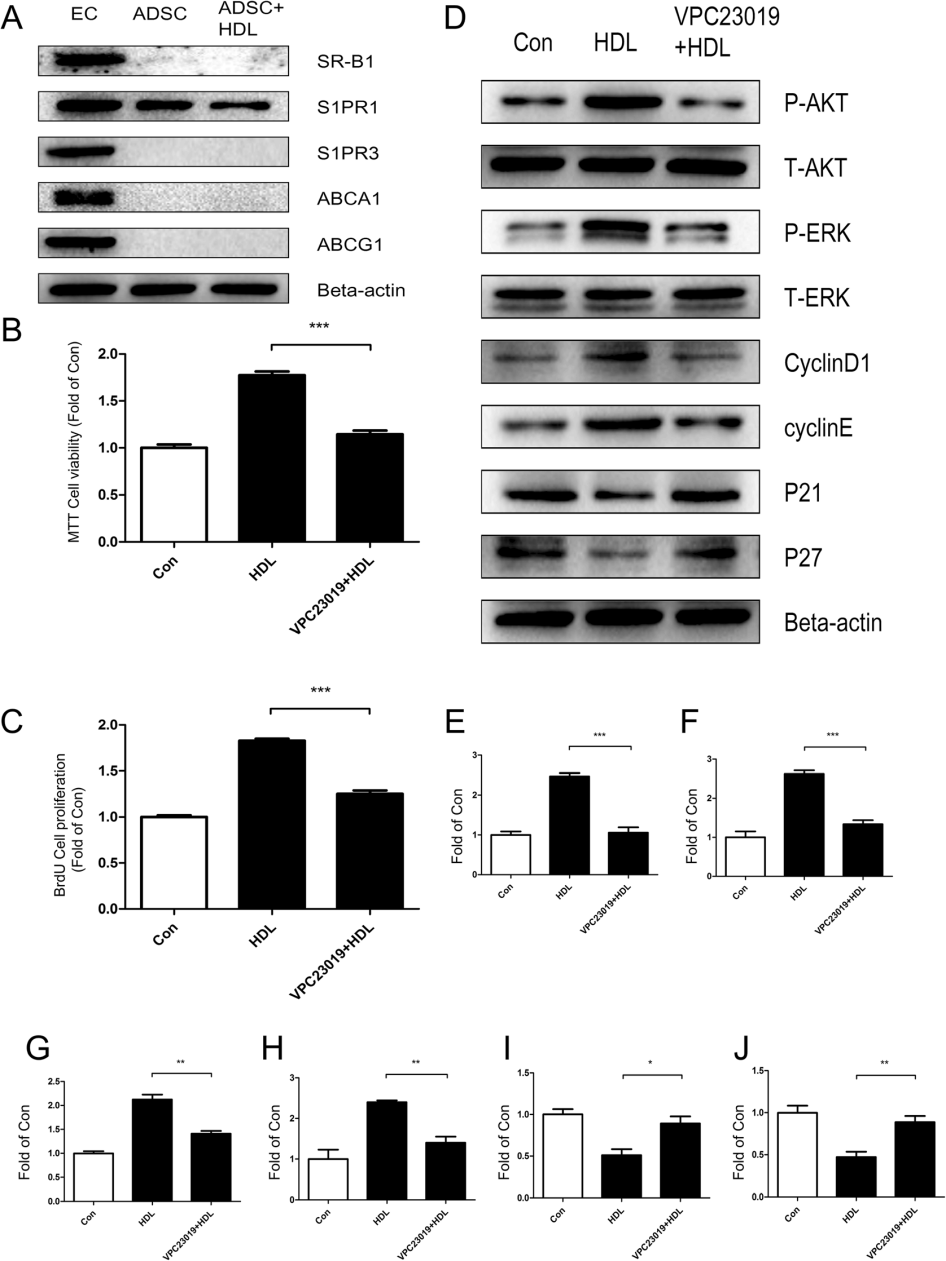
S1P1 receptor contributes to high density lipoprotein-induced cell proliferation of human adipose-derived stem cells. **a** Western blot confirms the presence of several classical receptors for high density lipoprotein (HDL) in human adipose-derived stem cells (ADSCs). Human umbilical vein endothelial cells (EC) were used as a positive control to validate the result. Data show that only S1P1, a classical receptor for HDL, was expressed in human ADSCs. **b** MTT assay and **c** bromodeoxyuridine (BrdU) assay show that the effect of HDL-promoting cell proliferation in human ADSCs was blocked when pretreated with S1P1 receptor specific inhibitor VPC23019 for 30 minutes prior to 24 hours of HDL treatment. **d** Western blot change in expression of various signal pathway molecules and cell cycle regulatory proteins in human ADSCs pretreated with S1P1 receptor inhibitor VPC23019 for 30 minutes prior to incubation with HDL for 1 hour. **e** p-Akt, **f** p-ERK, **g** cyclin D_1_, **h** cyclin E, **I** p21, **j** p27. Results are the mean ± standard error of three experiments for each condition determined from densitometry relative to corresponding total protein/beta-actin. *P <0.05, **P <0.01, ***P <0.001

We tested the expression of several classic HDL receptors on ADSCs. To our surprise, SR-BI [15], ABCA-1 [16] and ABCG-1 [16] were not expressed, while S1P1 receptor [17] from the family of S1P receptors was expressed (Figs. 6a and 7a). After pretreatment with S1P1 receptor inhibitor VPC23019, HDL-stimulated ADSC proliferation decreased significantly (Figs. 6b,c and 7b,c). The results of western blot analysis suggested that Akt and ERK1/2 signaling pathways are not activated, and the expressions of cell cyclin and CKI were not changed significantly compared with control. These results suggested that S1P1 receptors participate in HDL-induced proliferation of ADSCs and play an essential role proximal to Akt and ERK1/2 signals (Figs. 6d and 7d).

## Discussion

The exploration of stem cell transplantation-based therapies for various diseases including cardiovascular diseases is being carried out widely in the field of regenerative medicine. The major pitfall to all these therapies is the low survival and weak proliferation of the stem cells after transplantation, which unfortunately is the major determinant of therapeutic efficacy [7]. Although many attempts have been made to improve this pitfall, the complexity of the factors involved makes them difficult to overcome [18]. Since these transplanted cells enter the bloodstream first and then the microenvironment of tissues, altering specific circulating or local tissue factors could significantly improve the survival and proliferation rate of transplanted stem cells. Modification of these circulating or local factors could be a valuable step to improve the success rates of these therapies.

HDL not only plays an important role in cholesterol metabolism and cardiovascular disease [18], but also has antioxidant, anti-apoptotic and anti-aging effects on vessels *in vivo* [17]. There are many reports that confirm HDL can reduce the risk of the cardiovascular diseases. The effects of HDL on protecting vascular endothelial cells [19], and for promoting proliferation of vascular endothelial precursor cells [11] and bone marrow-derived stem cells [12], have been widely reported. Published literature suggests that phosphatidylinositol 3-kinase/Akt-dependent cyclin D_1_ activation plays an essential role in HDL-induced endothelial precursor cell proliferation [11], and that HDL promotes the proliferation of bone marrow-derived stem cells via activation of phosphatidylinositol 3-kinase/Akt and mitogen-activated protein kinase/ERK1/2 pathways. This well-established action of HDL raises the possibility of its potential effect on other exogenous stem cells which enter the body.

In previous reports, the proliferation of ADSCs was shown to be promoted by single growth factors such as epidermal growth factor, insulin-like growth factor-1 and fibroblast growth factor-2 [20] and also enhanced by a combination of multiple growth factors. Fibroblast growth factor-2 is a strong growth-stimulating factor and is essential for the long-term proliferation and self-renewal of ADSCs through the ERK1/2 signal pathway [21]. The proliferation of ADSCs can also be advanced by platelet-derived growth factor via Jun amino-terminal kinase activation [22], by sphingosylphosphorylcholine through activation of c-jun N-terminal kinase [23], and via oncostatin M by activation of the microtubule-associated protein kinase/ERK and the JAK/STAT1 pathways [24].

Our study found that isolated HDL from plasma has a significant role in promoting proliferation of ADSCs. HDL stimulates Akt and ERK1/2 phosphorylation and activates Akt and ERK1/2 signaling pathways in ADSCs. These two signal pathways involved in cell survival and cell proliferation have been widely reported in a large number of previous studies. Cell proliferation is tightly controlled by factors regulating cell cycle progression. The cell cycle progression is mainly regulated by three types of proteins: cell cyclins, cyclin-dependent kinases (CDKs) and CKIs, which have a role in negative regulation of cell cycle progression [25]. There are a number of cell cycle regulation sites, and the most classic site is G1/S phase transition regulation. Cyclin D binding to CDK4/6, and cyclin E binding to CDK2 jointly promote the G1/S phase progression. CKIs p21/p27 can bind to CDK2/4/6, exerting the function of blocking the effect of cyclin–CDK complexes, and thus preventing G1/S phase transition [25, 26]. Together, these classic types of proteins play essential roles in regulating cell cycle progression [26]. In this study, we find that HDL treatment increases cyclin D_1_/E expression of ADSCs, and reduces expression of p21/p27, thus promoting cell cycle progression.

To further illustrate the specific pathways through which HDL promotes proliferation of ADSCs, we examined the expression of several classic HDL receptors on ADSCs. Surprisingly, not only SR-BI [14] and ABCA-1 [27] but also S1P2 (data not shown) and S1P3 receptors were not expressed, while only S1P1 [15] was expressed. Inhibitors of S1P1 receptors diminished the effect of HDL on promoting ADSC proliferation, suggesting that S1P1 is the important receptor for HDL’s action in ADSCs. Sphingosine-1-phosphate receptors are G-protein-coupling receptors which include five members: S1P1, S1P2, S1P3, S1P4 and S1P5 receptors [28, 29]. S1P1 and S1P3 are the most widely expressed, whereas S1P2 is expressed in the brain, especially in white matter tract regions, and S1P4 and S1P5 expression is confined to blood vascular cells and the central nervous system, respectively. S1P1, S1P3 and S1P5 bind sphingosine-1-phosphate with high affinity, while lysophosphatidic acid preferentially binds to S1P2 and S1P4. The activation of S1P receptors can lead to activation of the small guanosine triphosphatase (GTPase) Ras and the ERK, resulting in cell proliferation [29]. S1P receptor signaling is also associated with the activation of phosphatidylinositol 3-kinase and Akt involved in cell survival and inhibiting apoptosis [30]. Activation of protein kinase C and phospholipase C are also attributed to S1P receptors, which can cause increasing free calcium in cytoplasm [31].

The major limitation of this study is that only one cell signaling pathway was explored based on prior literature, but other pathways could also be involved. S1P1 receptors have diverse effects on various signaling pathways and these pathways may be relevant in the ADSCs, which need to be explored systematically using high-throughput protein microarray technologies. Future studies will focus on confirming these exciting findings in an *in vivo* animal model using effective live cell markers tracked with sensitive *in vivo* signal monitoring equipment. This would need to be followed up with well-designed clinical trials exploring the effect of increasing HDL levels on the success of ADSC transplantation.

## Conclusions

**Fig. 8:**
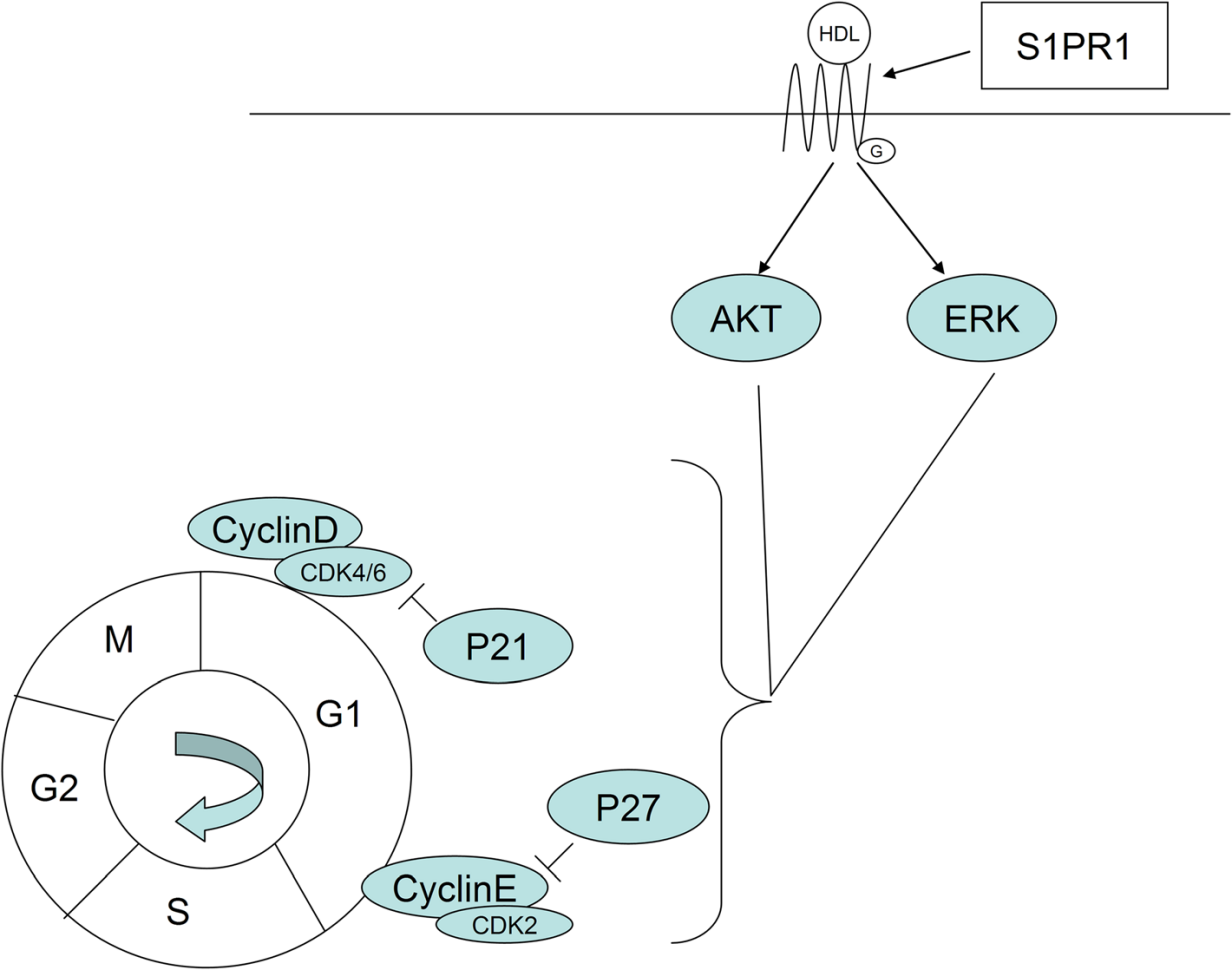
Hypothesized model for the molecular mechanism involved in high density lipoprotein promoting proliferation of adipose-derived stem cells. High density lipoprotein (HDL) binds to S1P1 receptor in the plasma membrane of adipose-derived stem cells, stimulates Akt and ERK1/2 phosphorylation, activates Akt and ERK1/2 signaling pathways in cytoplasm, increases expression of cell cyclins (cyclin D_1_ and cyclin E), and reduces the expression of cyclin-dependent kinase inhibitors (p21 and p27) to promote cell cycle progression and to achieve cell proliferation

In summary, this study is the first to explore the effects of HDL on ADSCs. Our results confirm that HDL can promote proliferation of ADSCs. HDL acts through the S1P1 receptor and activates Akt and ERK1/2 signaling pathways, thereby upregulating cyclin D_1_/E expression, and decreasing expression of CKIs p21 and p27, thus promoting cell cycle progression and proliferation (Fig. 8). This study raises the therapeutic option of raising levels of HDL to assist in improving post-transplant survival rate of ADSC, which needs to be further explored in future studies.

## Additional file

Additional file 1:
**is a figure showing flow cytometry data for CD29, CD90 and CD105 of mice ADSCs.**

## Abbreviations

ADSC: adipose-derived stem cell
BrdU: bromodeoxyuridine
CDK: cyclin-dependent kinase
CKI: cyclin-dependent kinase inhibitor
DMEM: Dulbecco’s modified Eagle’s medium
ERK: extracellular signal-related kinase
FBS: fetal bovine serum
HDL: high density lipoprotein
PBS: phosphate-buffered saline
PE: phycoerythrin
S1P1: sphingosine-1-phosphate type 1
