# Supplementary figures and images for: High density lipoprotein promotes proliferation of adipose-derived stem cells via S1P1 receptor and Akt, ERK1/2 signal pathways
Source: Stem Cell Res Ther. 2015 May 15;6(1):95. doi: 10.1186/s13287-015-0090-5 (PMC4453044; doi:10.1186/s13287-015-0090-5)

Supplemental Figure S1

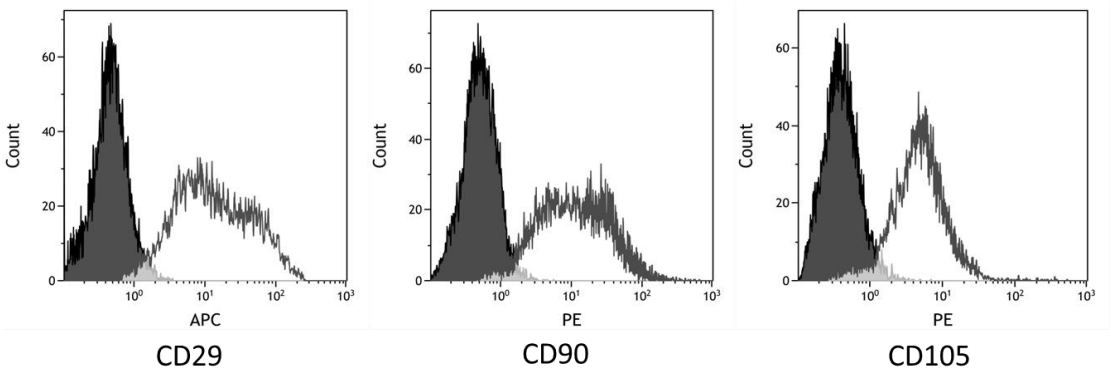

Supplement: Additional file 1: — is a figure showing flow cytometry data for CD29, CD90 and CD105 of mice ADSCs. [file 13287_2015_90_MOESM1_ESM.pdf]
